# Phytochrome Mediated Responses in *Agrobacterium fabrum*: Growth, Motility and Plant Infection

**DOI:** 10.1007/s00284-021-02526-5

**Published:** 2021-05-22

**Authors:** Peng Xue, Yingnan Bai, Gregor Rottwinkel, Elizaveta Averbukh, Yuanyuan Ma, Thomas Roeder, Patrick Scheerer, Norbert Krauß, Tilman Lamparter

**Affiliations:** 1 Botanical Institute, Karlsruhe Institute of Technology, 76131 Karlsruhe, Germany; 2 Zoological Institute, Molecular Physiology, Kiel University, Olshausenstrasse 40, 24098 Kiel, Germany; 3 German Center for Lung Research (DZL, Airway Research Center North), Kiel, Germany; 4 Present Address: Michael Smith Laboratories, Department of Microbiology and Immunology, University of British Columbia, Vancouver, BC Canada; 5 Institute of Medical Physics and Biophysics, Group Protein X-Ray Crystallography and Signal Transduction, Freie Universität Berlin and Humboldt-Universität Zu Berlin , Charité—Universitätsmedizin, Berlin, Germany; 6 Present Address: West China Hospital, Sichuan University, Chengdu, 610054 China

## Abstract

The soil bacterium and plant pathogen *Agrobacterium fabrum* C58 has two phytochrome photoreceptors, Agp1 and Agp2. We found that plant infection and tumor induction by *A. fabrum* is down-regulated by light and that phytochrome knockout mutants of *A. fabrum* have diminished infection rates. The regulation pattern of infection matches with that of bacterial conjugation reported earlier, suggesting similar regulatory mechanisms. In the regulation of conjugation and plant infection, phytochromes are active in darkness. This is a major difference to plant phytochromes, which are typically active after irradiation. We also found that propagation and motility were affected in *agp1*^−^ and *agp2*^−^ knockout mutants, although propagation was not always affected by light. The regulatory patterns can partially but not completely be explained by modulated histidine kinase activities of Agp1 and Agp2. In a mass spectrometry-based proteomic study, 24 proteins were different between light and dark grown *A. fabrum*, whereas 382 proteins differed between wild type and phytochrome knockout mutants, pointing again to light independent roles of Agp1 and Agp2.

**Supplementary Information:**

The online version contains supplementary material available at 10.1007/s00284-021-02526-5.

## Introduction

Soil bacteria of the genus *Agrobacterium* can transfer genes into plants and thereby induce the formation of plant tumors. This infection causes massive losses in agriculture, but the mechanism is on the other hand used for plant transformation in many research laboratories [1]. Tumor formation is dependent on the presence of a tumor inducing plasmid, the Ti-plasmid. The infection process starts with the excision of a single stranded sequence termed T-DNA from the Ti-plasmid. This T-DNA is transferred to the plant cell, where it is randomly inserted into the plant genome and where it induces the synthesis of auxin and cytokinin. These phytohormones stimulate tumor growth. The infected cells produce amino acid derivatives, opines, which are exported in the soil and can be used by *A. fabrum* for nutrition [2]. The entire virulence and infection process is induced by plant exudates such as acetosyringone; other environmental stimuli such as light are so far unknown. *A. fabrum* is known as soil bacterium [3] and it was long anticipated that it does not respond to light. However, sunlight penetrates several millimeters deep into the soil [4], and plant roots guide red light several centimeters into the soil [5]. In addition, *Agrobacterium fabrum* cells were also found on plant stems and leaves, *i.e.* in open sunlight environment [6]. For these reasons, it can be assumed that *A. fabrum* senses light, which is supported by the discovery of two phytochrome photoreceptors Agp1 and Agp2 in the sequencing of *A. fabrum* C58 genome [7, 8].

Phytochromes sense light in the blue, red and far-red range of the visible spectrum [9]. A large number of developmental effects are controlled by phytochromes in land plants [10] and fungi [11], and several examples for phytochrome effects in bacteria have been reported [12, 13]. In a search for phytochrome responses of *A. fabrum*, knockout mutants were generated which had, however, no apparent phenotype [14]. A first clear *Agrobacterium* phytochrome response was found by a computer based co-distribution study [15, 16]. In this investigation, we searched for Agp1 and Agp2 BLAST homologs in 43 related species that belong to the *Rhizobiales*. Then we tested for every other *A. fabrum* protein whether its BLAST homologs are similarly distributed among this set of species. Agp1 and Agp2 homologs were found in an almost identical subset of species as homologs of TraA, a central player in bacterial conjugation. Experimental tests showed that conjugation of *A. fabrum* is indeed regulated by light and phytochrome [15]. When strains with Ti plasmid were used, conjugation was drastically reduced by red or far-red light and in *agp1*^−^ or *agp2*^*−*^ knockout mutants. In the *agp1*^*−*^/ *agp2*^−^ double knockout, no conjugation was observed. Studies with complementation strains were more complex than expected but showed that the observed mutant effects were clearly the result of the loss of phytochrome.

We found here that phytochromes in *A. fabrum* have a major impact on plant infection. This response was also light regulated. Mutant results suggested an action of phytochrome in darkness, as in the control of conjugation. We also analyzed effects on *A. fabrum* growth and motility. In those cases, phytochromes could have an impact independent from light. Finally, the proteomes of dark and light grown wild type and double knockout mutants were compared. These data show that levels of few gene products are affected and suggest regulatory mechanisms independent from transcription or translation. On the level of single proteins, we found phytochrome effects that are light dependent and others that are light independent.

## Materials and Methods

### *Agrobacterium* Strains, Growth Conditions and Motility Assay

As wild type strain we used *Agrobacterium fabrum* C58 (former *Agrobacterium tumefaciens* C58). The name *A. fabrum* is used here because in protein and genome databases “*fabrum* C58” is used instead of “*tumefaciens* C58”, according to [17]. The authors are aware that this species name has never been validated (https://lpsn.dsmz.de/species/agrobacterium-fabrum). The phytochrome knockout mutants *agp1*^*−*^, *agp2*^*−*^ and the double knockout mutant *agp1/2*^*−*^ were generated by homologous recombination as described earlier [14]. These are the same strains that were used for conjugation and other experiments [18]. *Agrobacterium fabrum* was either grown in LB liquid medium or on 1% agar plates. For β-Glucuronidase (GUS) assays, strains with additional pGUSINT vector were used [15, 19]. For growth assays, 100 ml LB medium was first inoculated to achieve a cell density of *OD*_600 nm_ = 0.05 and kept at the desired temperature under shaking (110 rpm). The *OD*_600 nm_ was measured every day. Motility assays were performed on 10 cm LB agar plates with a reduced agar concentration of 0.5%. This concentration is between the 0.2 or 0.3% used to analyze flagella driven swimming [20] and the 1% used to observe type IV pili driven twitching motility [21]. The pH of the medium was adjusted by adding HCl or NaOH before the addition of agar and autoclaving. Eighteen dots of *A. fabrum* cells were spotted on each plate using a 100 µl pipette tip. Spot sizes were about 0.5 mm. The plates were brought into dark or light and incubated at the temperature given. After 30 h incubation the diameter of each colony was measured, the diameter was taken as value for motility. The mean value of the 18 diameters was calculated. Each treatment was repeated 3 times and the mean of the three mean values ± SE calculated.

### *Arabidopsis* Root Infection Assay

This assay was adopted from [22]. Seeds of *Arabidopsis thaliana* (ecotype Wassilewskija) were surface sterilized, suspended in 1 ml 0.1% agar solution, incubated over night at 4 °C, brought on agar-plates (B5-Gamborgs-Medium, Duchefa, Haarlem, Netherlands, 20 g/l sucrose, 7.5 g/l Bacto-Agar and 100 mg/l Cefotaxim) and kept at 20 °C under 16 h light/8 h dark cycles in an incubator (Percival Scientific, Inc., Perry, USA). After 20 d, roots were cut in 3–5 mm segments. 20 bundles of 5 roots were placed on each agar plate (4.3 g/l minimal salts, Duchefa, Haarlem, Netherlands, 0.05 M MES, 0.5 mg/l nicotinic acid, 0.5 mg/l pyridoxine, 0.5 mg/l thiamin, 100 mg/l myo-inositol, 10 g/l sucrose, 7.5 g/l Bacto-Agar, pH 5.7). *Agrobacterium fabrum* wild type and knockout mutants were kept for 3 days at 28 °C on YEP agar plates. 5 ml YEP liquid medium was inoculated with a single colony and incubated over night at 28 °C. A 50 ml culture was inoculated to a start *OD*_600 nm_ of 0.1 and cultivated under shaking until the *OD*_600 nm_ = 0.8. Cells were diluted 10× and transferred by centrifugation into 0.9% NaCl. Of each suspension, 15 µl were pipetted onto a root bundle. During 48 h the agar plates were kept at 26 °C in red (40 µmol m^−2^ s^−1^_,_ 655 nm) or darkness. After cocultivation, roots were brought on MS agar medium with 100 mg/l Cefatoxamin (to inactivate *A. fabrum*). These plates were kept for 2 weeks at 23 °C in darkness. The number of tumors was counted. Experiments were repeated 3 times independent of each other.

### Infection of *Nicotiana benthamiana* with *Agrobacterium fabrum*

*Nicotiana benthamiana* was grown for 6 weeks in the greenhouse. For stem assays, the surface of the stem was first cut at 1 to 3 positions with a sterile scalpel. Each cut was 1 cm long. *Agrobacterium fabrum* from agar plates was then applied directly onto the wounded sites using a pipette tip. For dark controls, sections were covered with aluminum foil, the other parts were left open. The entire plants were then kept in red light (1 µmol m^−2^ s^−1^, 655 nm) at 25 °C. After 24 h, aluminum foil was removed and the bacterial growth was terminated by pipetting 500 µl of 220 μM cefotaxime into each cut. Thereafter, the plants were kept at room temperature under daylight. After 6 weeks, tumors of the wound were observed and photographs were taken. For leaf assays, bacteria with the pGUSINT vector [19] were propagated in liquid LB medium at 28 °C until *OD*_600 nm_ reached 2. The GUS assay is described also in [1]. After centrifugation at 5000 g for 15 min, the supernatant was removed and the pellet was suspended in 11 mM MES, 10 mM MgCl_2_, pH 7 to the final *OD*_600 nm_ of 0.8. Two ml of this solution were infiltrated into a plant leaf from 6 week old plants as above by using a syringe without cannula. Thereafter, the plants were kept for 24 h in red light (1 μmol m^−2^ s^−1^) or in darkness. For GUS staining [23], the leaves were cut off and placed into X-Gluc staining solution containing 2 mM 5-bromo-4-chloro-3-indolyl glucuronide (X-Gluc, Thermo Fisher), 200 mM sodium phosphate (pH 7), and 0.01% (v/v) Triton X-100 at 37 °C for 17 h in darkness. After soaking the leaves in ethanol (first 70%, then 80%, then 90%, then 100%), the blue GUS stains were observed and photographed. For GUS fluororimetric assays, leaf extracts were incubated in reaction mix containing 50 mM sodium phosphate, 10 mM EDTA, 0.1% (w/v) SDS, 0.1% (v/v) Triton X-100, 1 mM 4-MUG (4-Methylumbelliferyl-β-D-glucuronide hydrate, Sigma), pH 7 for 17 h at 37 °C in darkness [23, 24]. The reaction was stopped by the addition of 1 M Na_2_CO_3_ to a final concentration of 0.99 M. The fluorescence was measured with a Jasco FP 8300 fluorimeter; excitation and emission wavelengths were 365 nm and 455 nm, respectively. Normalization of the GUS activity calculated as nmol of 4-MU per minute per leaf was performed using 4-MU with concentrations of 0.5, 5, 50, and 500 nM.

### Proteome Studies

Colonies of *A. fabrum* wild type or *agp1/2*^*−*^ double knockout mutant were used to inoculate 100 ml LB and the cultures were shaken overnight (28 °C, 110 rpm). The suspensions were diluted to an *OD*_600 nm_ of 0.6 and a volume of 100 ml. Half of the culture flasks were wrapped in aluminum foil (“darkness”). All culture flasks where shaken for 24 h under white light (40 µmol m^−2^ s^−1^). Two ml cultures were centrifuged at 12,000× *g* for 5 min at 4 °C. The cell pellets were washed three times with cold 10 mM Tris/Cl, 1.4 mM PMSF, 1 mM EDTA, pH 7.5 and frozen to − 80 °C. Subsequent steps were performed by the company Mtoz Biolabs (Mtoz Biolabs Inc, Boston, MA USA). About 80 mg frozen cells were suspended in 0.3 ml lysis buffer (50 mM Tris, 5% SDS, 0.1 mM EDTA, 150 mM NaCl, 1 mM MgCl_2_, 50 mM dithiothreitol, pH 8) [25] and homogenized. The supernatant was collected after centrifugation at 10,000× *g* for 5 min at 4 °C. The protein concentration was measured with Pierce™ BCA Protein Assay Kit (Thermo Fisher Scientific). Supernatant samples were diluted with 50 mM triethylammonium bicarbonate (TEAB) (pH 8.5) to a final protein concentration of 1 μg/μl (100 μl), reduced by 10 mM tris (2-carboxyethyl) phosphine (pH 7) at 56 °C for 1 h and alkylated by 20 mM iodoacetamide in darkness for 1 h at room temperature. After the addition of 600 μl cold acetone and incubation at − 20 °C, the mixture was centrifuged at 8000 g for 10 min at 4 °C. The acetone supernatant was removed and the pellet was dried for 2–3 min, reconstituted with 100 μl 50 mM TEAB (pH 8.5) and finally digested overnight at 37 °C by adding 2.5 μg trypsin (Madison, WI, USA). Peptides were labeled with tandem mass tags (TMT, Thermo Fisher Scientific Inc.) as listed in Table S1. Six samples from each group were mixed together for nanoscale liquid chromatography and tandem mass spectrometry (nano LC–MS/MS) analysis.

The nanoflow ultra high-performance liquid chromatography (UPLC) LC–MS/MS analysis was performed by a Dionex Ultimate 3000 Nano liquid chromatography (LC) system with Orbitrap Q Exactive™ mass spectrometer (MS/MS Thermo Fisher Scientific, USA) with an electrospray ionization nanospray source. LC was performed with an Easy-nLC1000 system (ThermoFisher Scientific, USA) equipped with a 100 μm × 10 cm in-house made nanocolumn, packed with a reversed-phase ReproSil-Pur C18-AQ resin (3 μm, 120 Å, Dr. Maisch GmbH, Germany). Of each sample, 5 μl were loaded into the nanocolumn. The mobile phase was 0.1% formic acid in water (A) and 0.1% formic acid in acetonitrile (B). The peptides were separated at a flow rate of 600 nl/min with LC linear gradient: 15 min, from 6 to 9% B; 20 min, from 9 to 14% B; 60 min, from 14 to 30% B; 15 min, from 30 to 40% B; 3 min, from 40 to 95% B; 7 min, 95% B. The MS parameters, resolution and precursor m/z range were set to 60,000 and 300–1650, respectively. The 15 most intense peptide ions from the MS scan were fragmented by collision-induced dissociation (40% normalized collision energy). We used the Orbitrap with a resolution of 15,000 for MS/MS scan. The raw MS dates were analyzed and searched with Proteome Discover 2.1 software (Thermo Fisher Scientific) against the protein database of *A. fabrum*. The entire procedure was repeated with 3 independent samples of each condition (wild type / mutant, light / dark).

## Results

### Growth in Liquid Medium

**Fig. 1:**
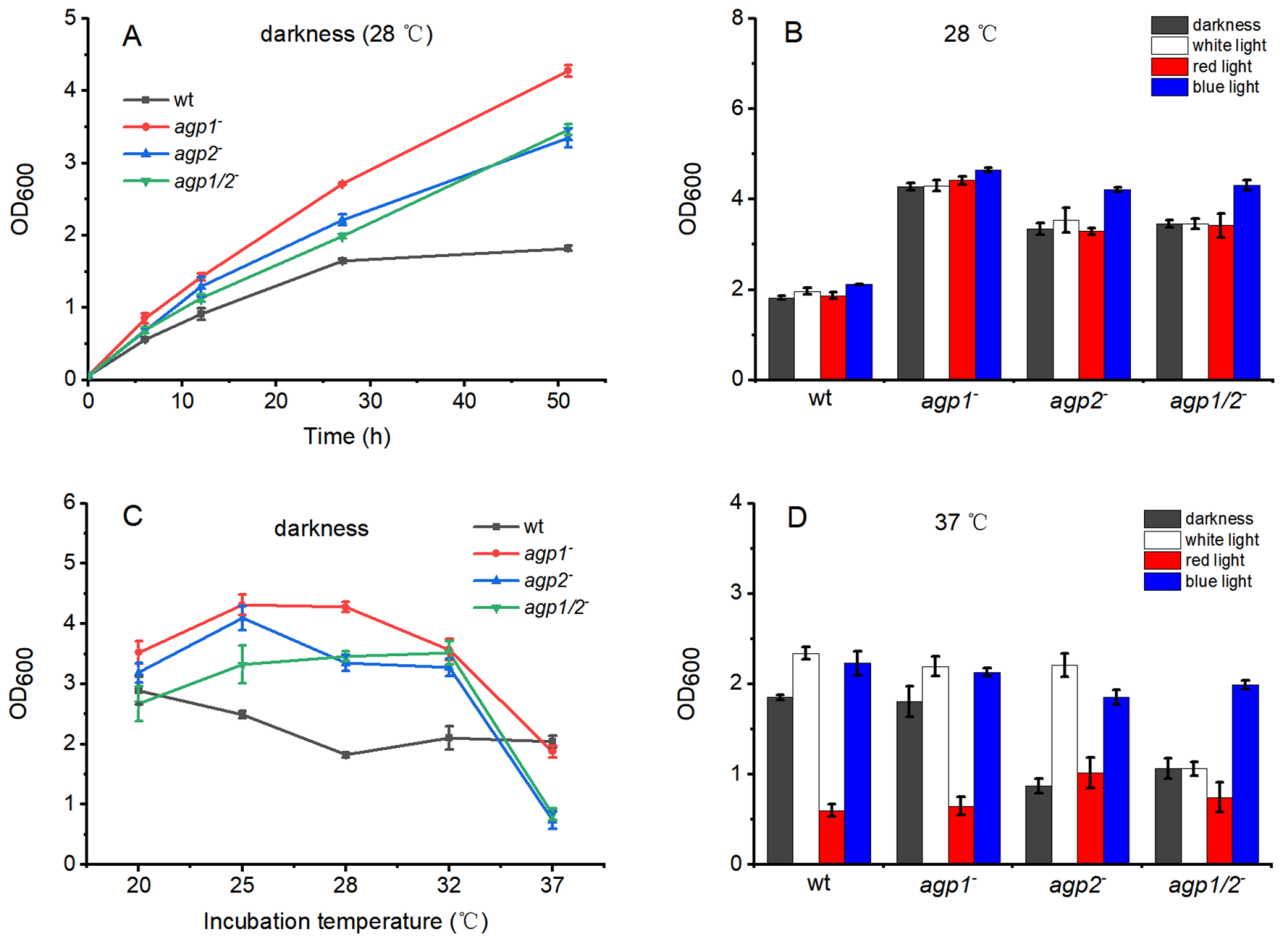
Effects of phytochromes Agp1 and Agp2 on growth of *A. fabrum* in liquid culture. **a** and **c** cell densities (OD_600_) plotted over time for wild type (wt) and phytochrome mutants of *A. fabrum* during growth in darkness at 28 °C (**a**) and 37 °C (**c**). Effect of white, red (655 nm), blue light (470 nm) and darkness on cell densities (OD_600_) of wild type and mutants of *A. fabrum*, 51 h after inoculation; cultivation at 28 °C (**b**) and 37 °C (**d**). Light intensities were always 40 µmol m^−2^ s^−1^. Mean values of three independent experiments ± SE

In earlier studies we found no effect of phytochromes on *A. fabrum* growth in liquid culture, but in these studies growth was followed for 6 h only [14]. In the present study, we expanded the growth period and performed assays at different temperatures, because Agp1 might also function as a thermosensor [15, 26]. The growth at 28 °C was not significantly different between wild type and mutants up to 6 h, as in the earlier studies, but thereafter, all three mutants displayed faster growth than the wild type (Fig. 1a, b). The *agp1*^*−*^ knockout strain had the fastest growth and reached 2× higher cell densities than the wild type at 51 h. These results show that Agp1 and Agp2 act inhibitory on growth in the later growth stage, i.e. under nutrient deprivation. That all three phytochrome mutants grow faster than the wild type makes it unlikely that the effects result from a second-site mutation. Agp2 seems to dominate the effect in the wild type, since the response of the double knockout mutant is comparable with the *agp2*^−^ and not with the *agp1*^−^ mutant. Despite the clear mutant effects, we observed no impact of light on the growth at 28 °C (Fig. 1b). At 37 °C, the cell densities of the *agp1*^*−*^ and *agp1*^*−*^/*agp2*^−^ mutants were lower as compared to the wild type and there was a clear inhibitory effect of red light on wild type and the *agp1*^*−*^ mutant. Therefore, red light regulation at 37 °C is mediated through Agp2. White light and blue light acted similarly. Both induced a slight increase in cell densities in wild type and *agp1*^*−*^ knockout and a strong increase in the *agp2*^*−*^ knockout. An induction of growth was induced by blue light in the double knockout (Fig. 1). *Agrobacterium fabrum* has no LOV or BLUF protein homolog, which could serve as alternative photoreceptors. The photolyase PhrB, which has been identified by a blue light effect on motility [27, 28] is probably the photoreceptor of these light responses in the absence of phytochromes.

### Cell Motility

An assay for cell motility or swimming of *A. fabrum* is based on the increase in colony diameter during a certain period of time. In earlier swimming plate studies, light reduced the motility of *A. fabrum* significantly; this effect led to the discovery of the (6–4) photolyase PhrB [29]. We performed here similar studies with the focus on phytochrome effects. When these motility experiments were performed on standard medium under different light conditions and with different knockout mutants, we found no clear light effect and no clear effect of the knockout mutations, in contrast to earlier studies [29]. We therefore expanded our conditions to a broad range of pH and to two different temperatures − 26 and 37 °C. Since spectral properties of Agp2 are pH-dependent [30] and Agp1 could act as thermosensor [26], it seemed reasonable to us that a possible impact of phytochrome and light on swimming could be uncovered by pH and temperature variations. An example of the variation in colony diameters at different pH is shown in Fig. S1. The whole set of data obtained at different pH, light, and temperatures is shown in Fig. S2. In most treatments, colony diameters of *A. fabrum* were smaller at pH 5 or pH 9–11 as compared to the neutral range of pH 6–8. In the neutral range, the single knockout mutants had slightly larger diameters than the wild type, whereas diameters of the *agp1*^−^*/agp2*^−^ double knockout were slightly smaller (Fig S2A). This points to a weak light independent effect of Agp1 and Agp2 on cell motility.

**Fig. 2:**
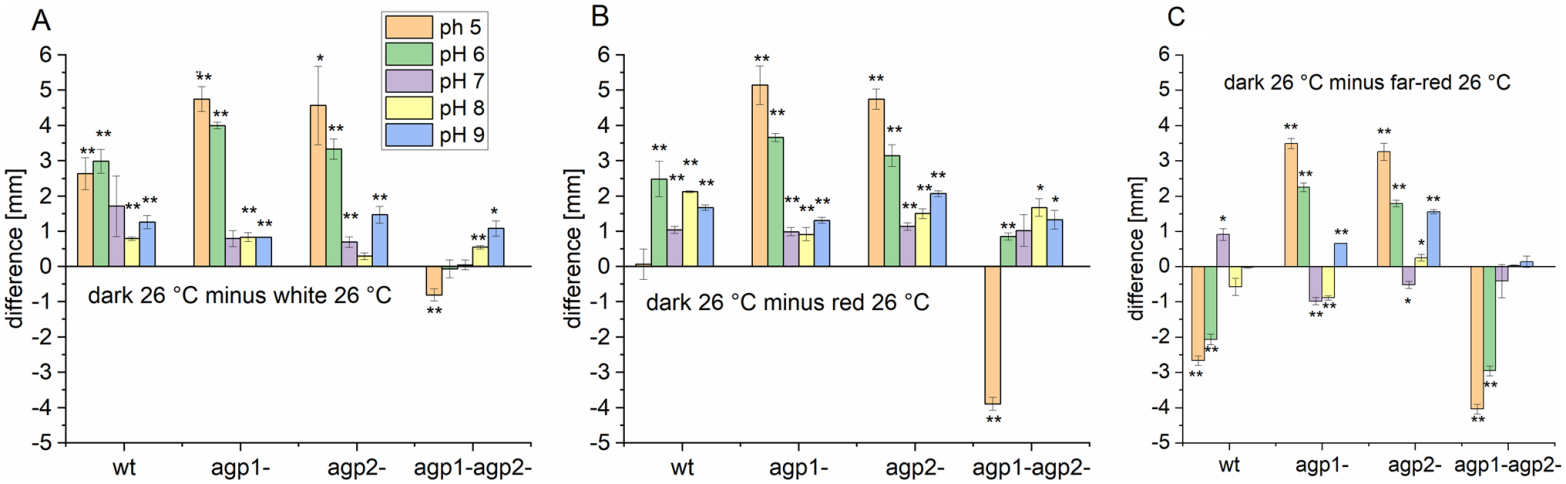
Light and mutant effects on cell motility of *A. fabrum* at different pH. Mean values ± standard errors (SE) of differences of colony diameters. **a** differences between darkness and white light, **b** differences between darkness and 655 nm red light, **c** differences between darkness and 780 nm far-red light. Colony diameters were determined 30 h after inoculation at 26 °C, light intensities were always 40 µmol m^−2^ s^−1^. The subtractions are based on the data presented in Fig. S2. Errors were calculated according to the laws of propagation of uncertainty. *T* test results for significant differences between dark and light treated samples are indicated by *(< 5% error probability) or **(< 1% error probability)

The dark-minus-light differences and the error bars of these differences together with t-test significances are shown in Fig. 2. Almost all colony diameters in white, red or far-red light were smaller than those in the dark, leading to positive difference values (Fig. 2a–c). The highest difference values, i.e. the strongest light effects were observed at pH 5. The white light effects were weaker than those of the earlier study [29]. White, red and far-red light resulted in a larger effect in the single knockout mutants as compared to wild type and double knockout (Fig. 2g, h). This suggests that each phytochrome acts also inhibitory on the light effect on motility, because changes are larger in absence of Agp1 or Agp2. In the double knockout, no light effects or stimulatory light effects (with negative difference values) were seen. This suggests that in the wild type, both phytochromes together act as photoreceptors for this light response. The “stimulatory light effect” was especially found for the far-red treated double knockout sample at pH 5 and pH 6. These are probably no outliers, as also other samples had a negative difference value (Fig. 2). Clearly, phytochromes cannot be photoreceptors for the stimulatory light response of the double knockout mutant (or for any light response). We are also not aware of another photoreceptor that could sense long wavelength red light in *A. fabrum*. We can only imagine that the negative response resulted from local warming induced by the light. The effect of local warming should as well take place in wild-type and single mutants. Since the inhibitory red/far-red light effects are antagonistic to the stimulatory effect, the proposed inhibitory role of both phytochromes in the wild type (Fig. 2a–c) is still valid.

The 37 °C experiments were performed in dark and white light only. In many conditions, the colony diameters were zero, *i.e.* the cultures died. Altogether, less data are available as for the 26 °C experiments. White light induced cell death at pH 9 in wild-type cells and at pH 9, pH 8 and pH 5 in single knockout mutants (Fig. S2F and G); the colony diameters were zero under these conditions. In the double knockout, no cell death was induced by white light. This result shows that the effect of light on cell death is mediated through phytochrome(s) in wild type and single knockouts.

### Plant Infection

**Fig. 3:**
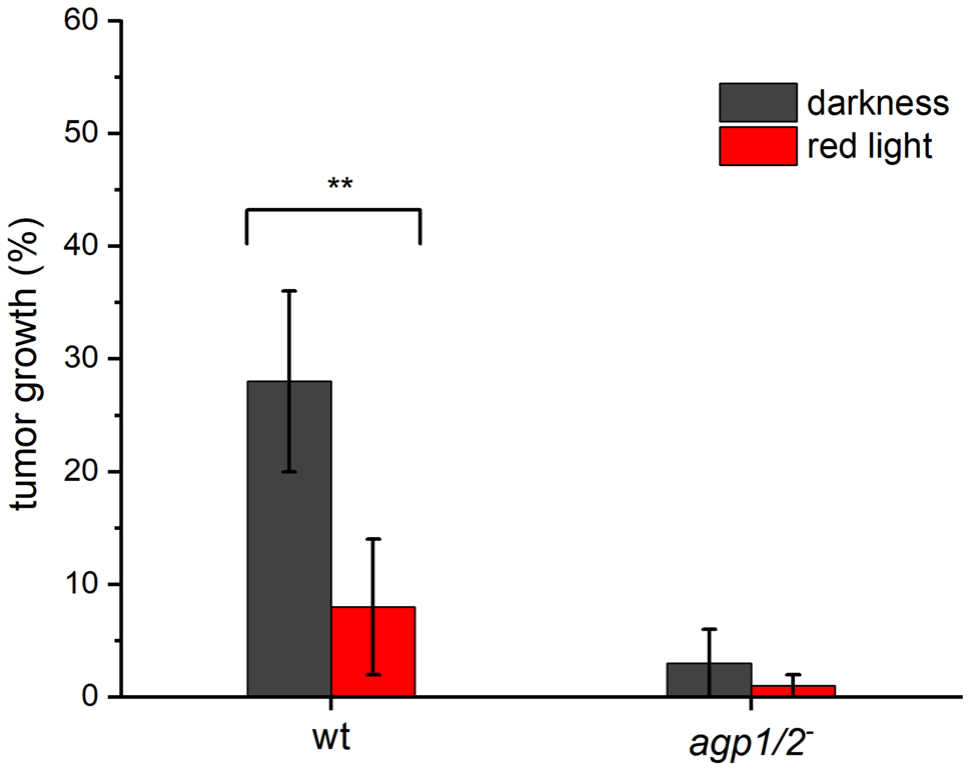
*Arabidopsis thaliana* root infection by *A. fabrum* wild type and *agp1/2*^*−*^ mutant under dark and red light (40 µmol m^−2^ s^−1^). *A. fabrum* suspensions were pipetted on root segment bundles and incubated for 2 days. The number of tumors was counted after 2 weeks. An example photo is shown in Fig. S1. Mean of 3 experiments ± SE

We analyzed *A. fabrum* light and phytochrome effects on virulence by root infection, stem infection and leaf infection assays. The root infection is monitored by tumor formation on root segments of *Arabidopsis thaliana* two weeks after cocultivation under different light (see Fig. S3 for tumor formation). We could perform only a limited number of experiments: the *Arabidopsis* roots must have appropriate size and age, and the plants must grow at constant temperature, since otherwise the variations are too large. We could obtain these conditions for experiments with *A. fabrum* wild type and the double knockout mutant in darkness and red light. The infection of *Arabidopsis thaliana* roots was clearly down-regulated by red light and in the *agp1*^*−*^/*agp2*^*−*^ double knockout mutant (Fig. 3). There was no light effect in experiments with the *A. fabrum* double knockout mutant. We therefore assume that induction of tumors is completely regulated through the *A. fabrum* phytochromes and that the plant light perception system does not play a role. The same argument holds for experiments on stem and leaf infection described below.

**Fig. 4:**
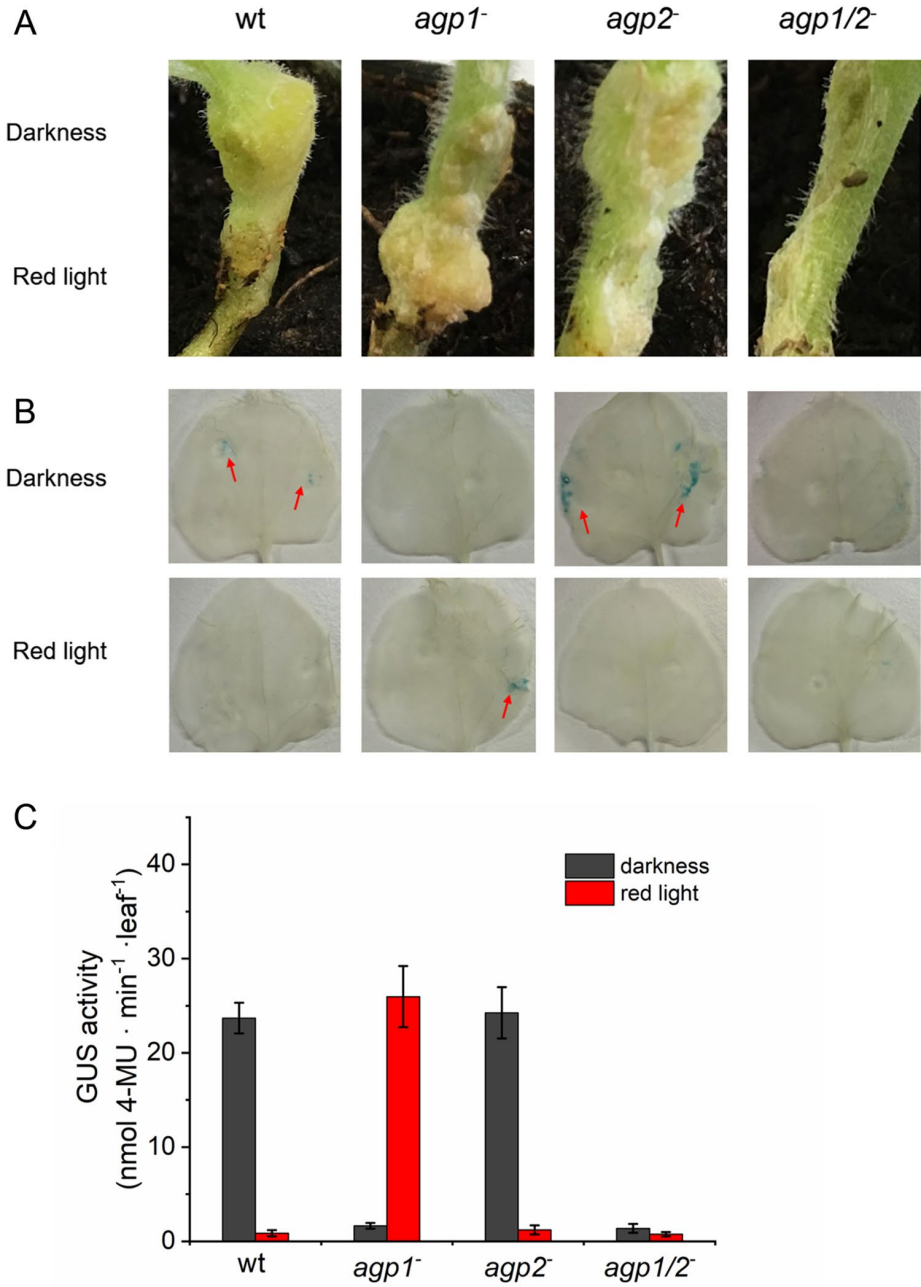
**a** Infection of *Nicotiana benthamiana* stems by *A. fabrum* wild type (WT) and phytochrome mutants as indicated above the panels. During 1 d infection, the upper part of the stem was covered with aluminum and the entire plant placed in red light (1 µmol m^−2^ s^−1^). Stems with or without tumors were photographed after 6 weeks. The experiments were repeated 3 times with similar outcome. **b** *Nicotiana benthamiana* leaves were infected with *A. fabrum* WT and phytochrome mutants (*agp1*^*−*^, *agp2*^*−*^ and *agp1/2*^*−*^) and the GUS activity stained with X-Gluc. **c** Quantification of leaf infection assay by MUG assay. Mean values of 3 independent infected leaves ± SE

Stem infection assays were performed with *Nicotiana benthamiana* plants (Fig. 4a). In this case, we assayed also tumor formation. *A. fabrum* cells were transferred to 2 injured sections on the same plant above each other. For a dark control, one section was covered with aluminum foil. Plants were kept for 1 day in red light, thereafter the aluminum foil was removed and bacterial cells were destroyed. After two weeks the formation of tumors could be observed in the dark controls of wild type or *agp1*^*−*^ infected stems, but not in the parts of the stems that were exposed to red light during infection. The results were reversed for the *agp2*^*−*^ mutant, in this case larger tumors were formed in the light and weak tumors in darkness. When the *agp1*^*−*^/*agp2*^−^ double knockout was used for stem infection, there were no tumors or only small tumors formed both in light and darkness. After plant growth of 6 weeks, tumors were increased but the differences between the different treatments remained (Fig. 4a). Note that here and in the root infection experiments (Fig. 3) the major difference between *A. fabrum* wild type and phytochrome double knockout is observed in darkness, i.e. indicates a dark action of phytochromes.

*Nicotiana benthamiana* leaf infection assays were monitored by glucuronidase (GUS) expression. For these assays, *A. fabrum* wild-type and phytochrome mutants with the pGUSINT vector [15, 19] that induces expression of GUS in infected cells (Fig. 4b, c), were used. X-Gluc results in a blue staining of these cells (Fig. 4b). With wild type *A. fabrum,* cells were infected after dark incubation but not after incubation in red light. When leaves were infiltrated with *agp1*^*−*^/*agp2*^−^ double knockout mutant cells, no staining was observed. Infiltration with *agp1*^−^ knockout mutants resulted in staining in the red irradiated leaf, but not in the dark control. With the *agp2*^−^ knockout mutant, the result was again reversed: cells were stained after dark incubation but not after red light treatment.

For better quantification, this assay was also performed with methylumbelliferyl-β-d-glucuronide (MUG) [24]. These measurements confirmed the X-Gluc results. After treating the leaves as above, leaf extracts were mixed with MUG and the fluorescence of the GUS product methylumbelliferol (MU) was measured. The MU signal was again strong in the dark control, whereas red light resulted in very low MU levels. With the *agp1*^*−*^/*agp2*^−^ double knockout the MU signal was always low. The light/dark pattern of the *agp1*^−^ mutant was again similar as the wild type and the pattern of the *agp2*^*−*^ knockout mutant again reversed.

All infection assays show that the gene transfer from *A. fabrum* to plant is controlled by light and by both phytochromes. In both kinds of DNA transfer –conjugation and plant infection—the transfer is efficient in darkness and suppressed in the light. Simply speaking, phytochromes are active in darkness and inactive in the light. The presence of Agp1 and Agp2 is required for most efficient gene transfer. A plant phytochrome or other plant photoreceptors could also mediate light effects on plant infection, but the light effects seen here are clearly mediated by Agp1 and Agp2.

### Proteome Studies

**Fig. 5:**
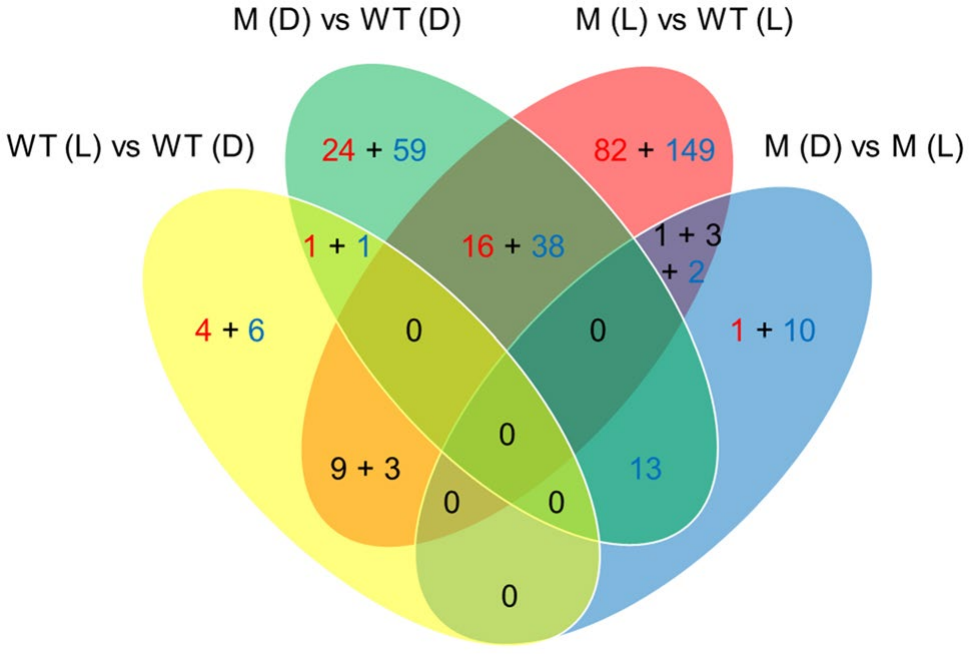
Differentially expressed proteins as identified by TMT analysis, Venn diagram. WT (D): wild type (darkness); WT (L): wild type (white light); M (D): mutant *agp1/2*^*−*^ (darkness); M (L): mutant *agp1/2*^*−*^ (white light). Ratio (experimental group versus control group) > 1.5 (*P* < 0.05) and < 0.67 (*P* < 0.05) were set as significantly up-regulated (red numbers) and down-regulated (blue numbers), respectively. The black numbers 9 + 3 indicate that 9 proteins were down-regulated in WT (L) versus WT (D), and up-regulated in M (L) versus WT (L) and 3 proteins were up-regulated in WT (L) versus WT (D) and down-regulated in M (L) versus WT (L). The black numbers 1 + 3 indicate that 1 protein was up-regulated in M (L) versus WT (L) and down-regulated in M (D) versus M (L) and 3 proteins were down-regulated in WT (L) versus WT (L) and up-regulated in M (D) versus M (L). The sum of differentially expressed proteins and identified protein was 422 and 2812, respectively. 134 and 353 proteins were only observed in both WT (D) and M (D) and both WT (L) and M (L), respectively

In order to find light versus dark or mutant versus wild type differences on the level of proteins, we performed a comparative TMT proteome analysis [31]. In this approach, proteins from cell extracts are digested by Trypsin and covalently labeled with tags of slightly different weight to mark each specific sample (Table S1). After labeling, samples are mixed and subjected to LC–MS/MS. The quantity of each peptide relative to the same peptide of another sample can be gained from comparison in the same run. The analysis was performed with 3 independent extracts of 4 different samples, wild type, double knockout both in dark and light. Out of 5400 *A. fabrum* proteins, 2814 were identified. We considered protein ratios of < 0.67 or > 1.5 with t*t* test probabilities < 0.05 as significant. An overview of the differences is given in the Venn diagram in Fig. 5. Of the 2814 detected proteins, 422 proteins were either light regulated or affected by the knockout mutation. In the wild type, 24 proteins appeared light regulated (Table S2 and S3). Ten of the 24 proteins showed light / dark differences in the wild type only, but not in the double mutant (Table S2). Seven light regulated proteins have functions in energy metabolism, three are ribosomal proteins, two are signal transduction proteins, one is a diguanylate phosphodiesterase and one a Ras family protein. On this level, no overlap with the physiological functions above is apparent. Quite interestingly, 24 proteins were light regulated in the double knockout mutant and not in the wild type. These effects could also be mediated by the photolyase PhrB, as discussed above for physiological responses.

More differences were found between the mutant and wild-type strains. Altogether, 382 proteins had a different abundance in mutant and wild type (Fig. 5). These results suggest that besides their roles as photoreceptors, phytochromes have also other functions both in darkness and in light, in line with the above phytochrome effects on growth.

In the following, we focus on proteins related to motility, conjugation, plant infection and type IV secretion, for which phytochrome effects have been found.

#### Motility

Out of 8 detected chemotaxis proteins, one (McpC) showed 0.4-fold ratio between mutant and wild type, the others were not affected by light or mutant (Table S4). Of the 34 flagella proteins, 11 were detected in the assay (Table S5). For 8 of these, no significant differences between dark versus light or mutant versus wild type were detected. For FlaA and FlaB, the major constituents of the bacterial flagella, the protein levels in the mutant were ca. 1.7 fold higher as compared to the wild type.

The type IV pili might contribute to movement on agar surface. In the present assay, 5 out of 10 type IV pili proteins were detected (Table S6). Two of these, CtpA and CtpE, revealed differences between mutant and wild type. CtpA of light samples was ca. 1.5 fold higher in the mutant, CtpE levels were 0.5 times lower in the mutant, both in light and dark samples.

#### Conjugation

Four of 22 proteins that are related to bacterial conjugation were detected in the assay (Table S7). All four proteins revealed differences between mutant and wild type. The central conjugation protein TraA cleaves the plasmid DNA, forms a covalent link with the DNA and unwinds the double stranded DNA. *Agrobacterium fabrum* has three TraA proteins that are encoded on the circular chromosome, the AT-plasmid and the Ti-plasmid [15]. Of these, only the AT-plasmid encoded TraA (Atu5111) was detected and the detection was only possible for dark samples. This points to light regulation of this TraA by phytochrome. The levels of the mutant were about 3 times lower as of the wild type.

#### Virulence

Of the 26 proteins assigned to plant infection or virulence, only 2 were detected (Table S8), VirH1 and AcvB. Levels of both proteins were unchanged in mutant versus wild type or dark versus light.

#### Type IV Secretion System

The type IV secretion system is important for both conjugation and plant infection. Eight of 26 proteins were detected (Table S9). AvhB1 was different between wild type dark and wild type light and three others, AvhB4, AvhB9 and AvhB10, were different between mutant and wild type.

#### Type VI Secretion System

Through type VI secretion system, toxin proteins are injected in competing bacterial cells [32]. We found two proteins with wild-type vs. mutant differences (Table S10) that belong to the type VI secretion system. The levels of the secretion protein Hcp were lower in mutant than in wild-type cells (light and dark). One toxin protein, Atu4347, annotated as peptidoglycan amidase, had lower levels in the mutant (light) as compared to the wild type (light). These proteins are involved in interbacterial competition. Preliminary experiments suggest indeed that phytochromes could play a role in interbacterial competition (data not shown).

## Discussion

We provide evidence for the involvement of bacterial phytochromes in the regulation of plant infection, in motility and growth in liquid culture. The regulation of bacterial conjugation by phytochromes has been reported in an earlier study [15]. The present proteome studies provide us with further information on phytochrome effects in *A. fabrum.*

The phytochrome mediated regulation of plant infection by light can be regarded as the most important finding of the present work. The mechanism of DNA transfer has been studied intensively and is used by many botanical groups for plant transformation [33]. Our data show that we indeed observe phytochrome responses of *A. fabrum* and not effecte that are mediated by the plant or other factors [34], because of the major difference between the *A. fabrum* double knockout mutant and the wild type. That all three plant infection effects under investigation—root-, stem- and leaf-infection—have a similar pattern with respect to light and dark differences and wild type and mutant differences makes us confident that we observed true *A. fabrum* phytochrome effects.

Plant infection and conjugation (in presence of the Ti plasmid) [15] are high in the dark and low or zero in the light. In the wild type, phytochromes could act on these effects either inhibitory in the light or stimulatory in darkness. The double knockout results show that phytochromes must be active in the dark: the loss of both phytochromes results in a loss of the effect in darkness. We would like to stress here that this dark action of phytochrome is a major difference between *A. fabrum* and the general phytochrome regulation pattern of plants. In principal, plant phytochromes have no dark activity, as concluded from mutant studies: dark grown phytochrome mutant seedlings are usually not different from wild-type seedlings [35]. In a publication by Hangarter et al. [36], evidence for dark activity of phytochrome was provided, but later discussed as second-site effect of the mutant. Earlier, our group has found evidence for dark activity at elevated temperature [37]: Dark grown *Arabidopsis* phytochrome B mutant seedlings grew shorter than the wild type at 32 °C. To our knowledge, this is however the only report for a dark action of plant phytochromes, we must assume that generally, plant phytochromes are only active after illumination. Fungal phytochromes on the other hand do reveal clear dark activity [38]. A common feature of fungal and bacterial phytochromes like Agp1 and Agp2 is the biliverdin chromophore, whereas plants have a phytochromobilin chromophore. Fungal and bacterial phytochromes are light regulated histidine kinases, whereas plant phytochromes have lost their histidine kinase activity. Indeed, the histidine kinase activity of Agp1 is strong in darkness and down-regulated in the light [37], in agreement with the dark activity of Agp1 in conjugation and plant infection. In addition to the dark activity of phytochromes in *A. fabrum*, we also distinguished between light-dependent effects, such as conjugation and plant infection, and light independent phytochrome effects, such as growth (Table 1). The majority of protein differences on our proteome study belongs to the light independent effects.

**Table 1:** Summary of phytochrome effects in *A. fabrum*

	Wild type		*agp1*^-^ knockout		*agp2*^-^ knockout		*agp1*^*−*^/*agp2*^*−*^ double knockout		
	Dark	Light	Dark	Light	Dark	Light	Dark	Light	Dark action of Agp1 or Agp2
Light dependent phytochrome effects									
Motility at 26 °C	Normal	Normal	Normal	Lower	Normal	Lower	Normal	Normal	
Motility at 37 °C	Normal	Normal	Normal	Dead at extreme pH	Normal	Dead at extreme pH	Normal	Normal	
Conjugation	Normal	Low	Low	Very low	Low	Very low	Zero	Zero	Yes
Root infection	Normal	Low	n.d	n.d	n.d	n.d	Low	Very low	Yes
Stem infection	Normal	Low	Low	Normal	Normal	Low	Very low	Very low	Yes
Leaf infection	Normal	Very low	Very low	Normal	Normal	Very low	Very low	Very low	Yes
Light independent phytochrome effect									
Growth at 28 °C	Normal	Normal	High	High	High	High	High	High	Yes
Growth at 37 °C	Normal	Normal	Normal	Normal	Low	Normal	Low	Low	Yes

**Fig. 6:**
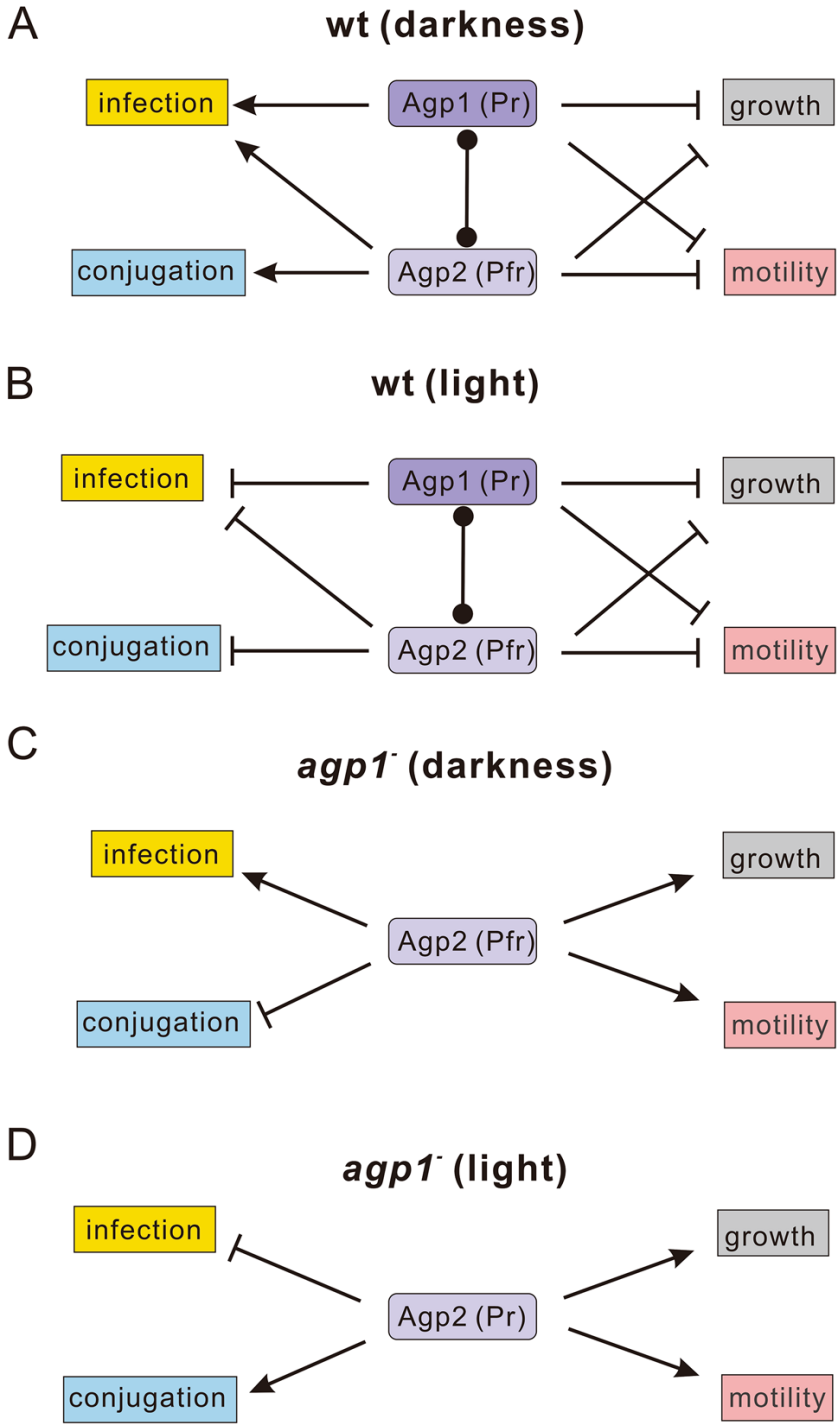
Possible model of phytochrome responses in *A. fabrum*. The model includes effects on conjugation, infection, growth and motility. Four scenarios are shown: wild type (**a** and **b**) and mutant *agp1*^−^ (**c** and **d**) in darkness (**a**, **c**) and light (**b**, **d**). The model is based on the interaction between Agp1 and Agp2 [39]

The *agp1*^*−*^ mutant pattern on plant infection is paradox: in this mutant, the light/dark pattern is reversed (Fig. 4), whereas in the *agp2*^*−*^ mutant, the light / dark pattern is like that of the wild type. A reversion of light regulation is found both in stem infection and in leaf infection assays. Such a reversion must be based on both inhibitory and inductive action of phytochromes, and could be related to an interaction of both phytochromes. We have shown earlier that Agp1 and Agp2 interact with each other physically and that spectral properties and autophosphorylation are modulated by this interaction [39]. For a further detailed understanding of this paradox result, more mutant studies and molecular studies are required. A simple model for the action of both phytochromes on conjugation, infection, growth and motility and the reversion in the *agp1-* mutant is provided in Fig. 6.

Conjugation and plant infection have in common that both are connected with DNA transfer in which the type IV secretion system is involved. In both cases, plasmid DNA is nicked by a relaxase at a defined position. In both cases, a single strand is covalently bound to the protein, DNA is unwound in a helicase-catalyzed reaction and the single stranded DNA–protein complex transported into the target cell. Quite interestingly, 4 out of 4 conjugation proteins that were detected in the TMT assay were different between wild type and double knockout mutant. In case of the type IV secretion system, these are 3 out of 8. Regulation of protein concentration could be one mechanism in the phytochrome regulation of DNA transfer. In mRNA microarrays there was no evidence for light or phytochrome regulation of transcription of any of these proteins (data not shown). Therefore, we consider a regulation of protein degradation or protein stability as cause for these differences. Since the differences in protein abundance are not large, and there is no evidence for an impact of light on these protein levels, we consider however different mechanisms of signal transmission of light regulation. In conjugation and plant infection, dark action of phytochrome is reduced in the light. This pattern correlates with the histidine autokinase activity of Agp1 [7], but not with that of Agp2 [39]. Phosphotransfer is thus a possible initial signal transmission mechanism, but cannot be the only one. We have recently shown that Agp1 and Agp2 do interact in vitro [39]. Such an interaction could partially explain the co-action of both phytochromes that is found in most effects. Conjugation and plant infection could be mediated through a direct interaction of Agp1 and Agp2 with VirD2 and TraA, proteins that catalyze the first steps in DNA transfer processes of plant infection and conjugation, respectively, and modulate in a light dependent manner the nuclease activities of the enzymes.

Growth of *A. fabrum* (at ambient temperature) is phytochrome regulated in a light independent manner (Table 1). In the proteome studies, the majority of regulated proteins was found in mutant versus wild type comparisons, again indicating a light independent impact of phytochromes. “Light independent” and “dark active” are similar phenomena. A light independent effect is probably an effect where phytochrome is active both in the dark and in the light. Therefore, the list of proteins that were identified as phytochrome dependent but light independent can add up to the list of the other dark effects.

Specifically in the dark, phytochromes stimulate conjugation and infection, but inhibit growth and motility. Apparently, phytochromes function to direct metabolism toward synthesis of gene-transfer proteins, away from growth and motility. The increase of growth in phytochrome mutants could be a direct result of the inhibition of conjugation or infection.

The present study, together with earlier work, shows that both DNA transfer processes of *A. fabrum*, conjugation and plant infection, are controlled by light and by phytochromes. Based on microarray and proteome studies, we exclude differential gene activation as a first step of phytochrome regulation and propose a direct modulation of proteins that are involved in the first steps in DNA transfer processes.

## Supplementary Information

Below is the link to the electronic supplementary material.

Supplementary file1 (DOCX 349 kb)

## Data Availability

All data are available, material upon request.
