# Supplementary material for: Phytochrome Mediated Responses in Agrobacterium fabrum: Growth, Motility and Plant Infection
Source: Curr Microbiol. 2021 May 22;78(7):2708–19. doi: 10.1007/s00284-021-02526-5 (PMC8213605; doi:10.1007/s00284-021-02526-5)
Supplement: Supplementary file 1 — Supplementary file1 (DOCX 349 kb) [file 284_2021_2526_MOESM1_ESM.docx]

**Phytochrome mediated responses in *Agrobacterium fabrum*: growth, swimming, plant infection** **and interbacterial competition**

Peng Xue, Yingnan Bai, Gregor Rottwinkel, Elizaveta Averbukh, Yuanyuan Ma, Thomas Roeder, Patrick Scheerer, Norbert Krauß, Tilman Lamparter

**Supplementary Figures and Tables**


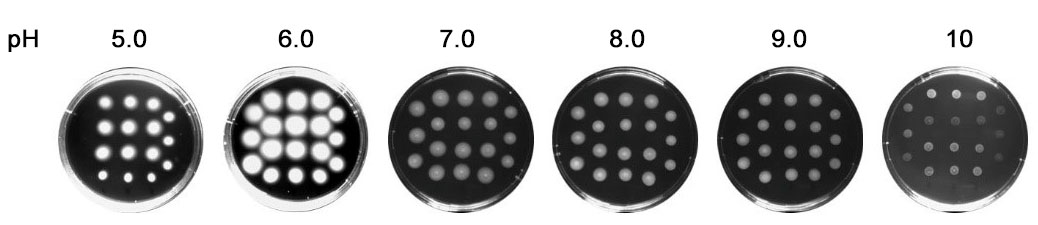


**Supplementary Figure S1.** Example for *A. fabrum* swimming assay; wild type on 0.5% LB solid medium with different pH 30 h after inoculation


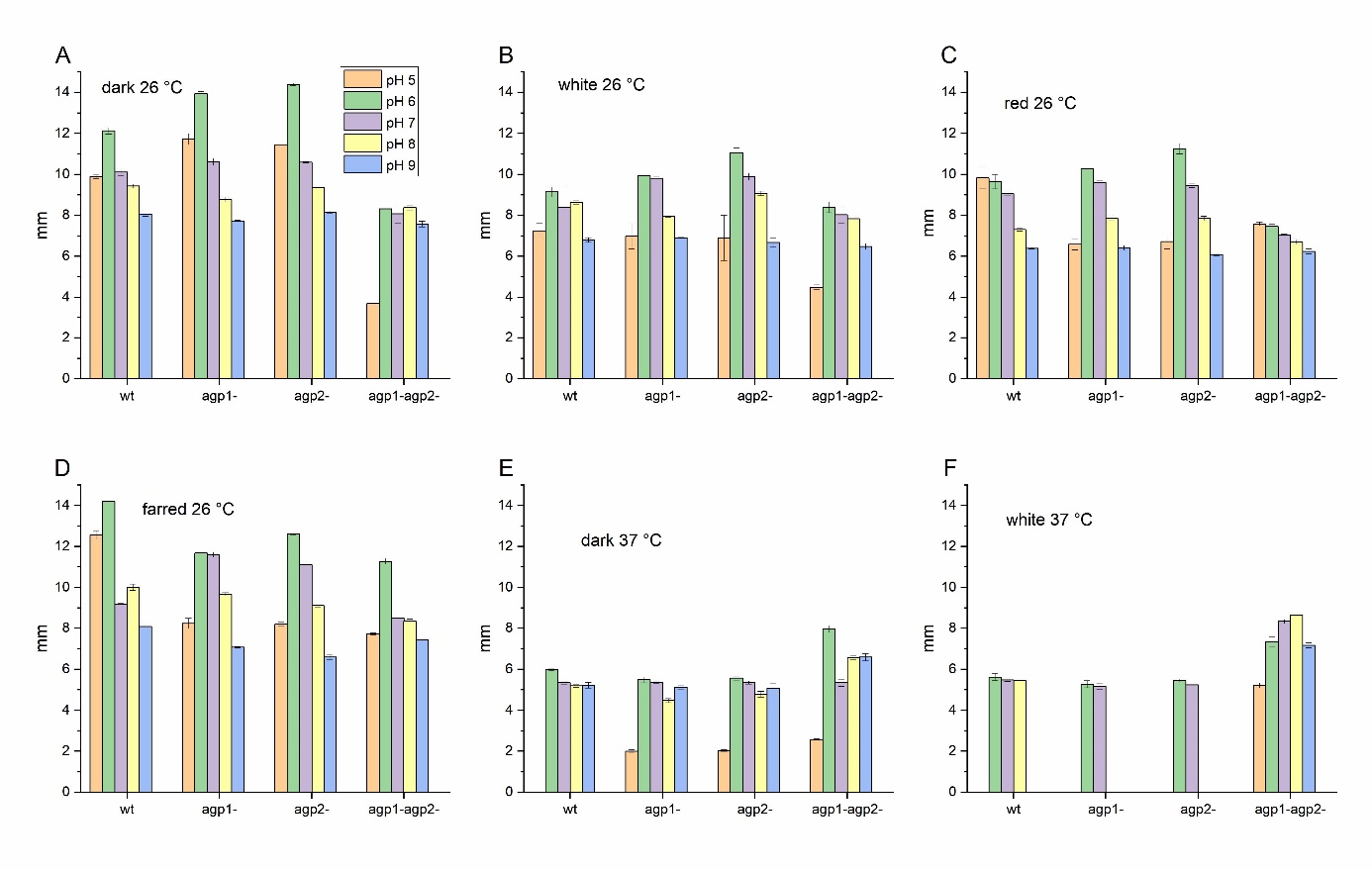


Figure S2 Cell motility of *A. fabrum*. The colony diameters were determined 30 h after inoculation at the indicated temperature and light conditions. Light intensities were always 40 µmol m^-2^ s^-1^. For each treatment, 18 colonies were inoculated under identical conditions, each treatment was repeated another two times in the same week and with the same start culture (stored at 4° C), so that altogether 3 repetitions were performed. The mean values and SE of all treatments is presented.


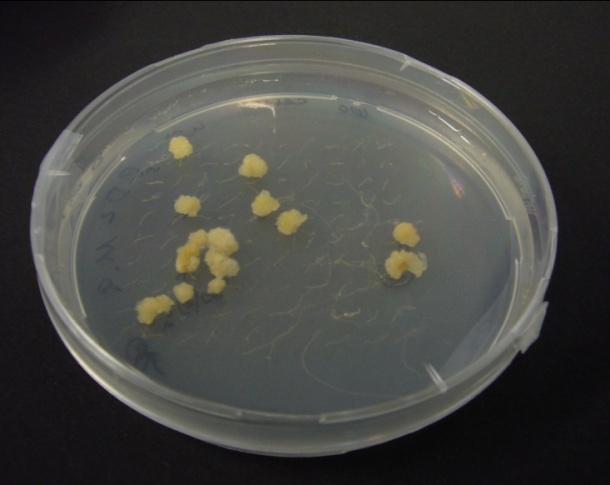


**Supplementary Figure S3.** Tumors of *Arabidopsis thaliana* roots 2 weeks after infection with *A. fabrum,* examples for root infection assay.

**Supplementary Table S1.** Sample labeling information. D: darkness; L: white light; WT_D: wild type_darkness; WT_L: wild type_white light; M_D: mutant_darkness; M_L: mutant_white light

| Group ID | Sample Name | Label Reagent |
| --- | --- | --- |
| D | M_D1 | TMT10-126 |
| D | M_D2 | TMT10-127N |
| D | M_D3 | TMT10-127C |
| D | WT_D1 | TMT10-128N |
| D | WT_D2 | TMT10-128C |
| D | WT_D3 | TMT10-130N |
| L | M_L1 | TMT10-126 |
| L | M_L2 | TMT10-129N |
| L | M_L3 | TMT10-129C |
| L | WT_L1 | TMT10-130N |
| L | WT_L2 | TMT10-130C |
| L | WT_L3 | TMT10-131 |

**Supplementary Table S2. iTRAQ/TMT assay,** ratios between dark and light for 10 proteins which are significantly higher or lower in dark vs. light of wild type, no significant L/D difference in double knockout (see yellow field in Venn diagram Fig. 6)

| Protein | Function | Fold change >1.5 or < 0.67; *P* < 0.05 |
| --- | --- | --- |
| 6 proteins | | |
| Rho (Atu2833) | Transcription termination factor | WT(D) / WT(L)=1.56 |
| ClpP (Atu1258) | ATP-dependent Clp protease proteolytic subunit 2 | WT(D) / WT(L)=1.67 |
| NuoI (Atu1278) | NADH-quinone oxidoreductase subunit I | WT(D) / WT(L)=1.59 |
| RpsL (Atu1951) | 30S ribosomal protein S12 | WT(D) / WT(L)=1.82 |
| AtpI (Atu0713) | ATP synthase protein I | WT(D) / WT(L)=1.85 |
| Atu6048 | putative DEAD/DEAH box helicase | WT(D) / WT(L)=1.56 |
| 4 proteins |  |  |
| CobL (Atu2798) | Precorrin-6y methyltransferase | WT(D) / WT(L)=0.67 |
| Atu2197 | putative diguanylate phosphodiesterase | WT(D) / WT(L)=0.51 |
| Atu4876 | Putative oxidoreductase | WT(D) / WT(L)=0.57 |
| Atu6114 | putative Ras family protein | WT(D) / WT(L)=0.63 |

**Supplementary Table S3. iTRAQ/TMT,** ratios between dark and light for 13 proteins which are significantly higher or lower in dark vs. light of wild type and the agp1- agp2- double knockout mutant. See yellow and red overlapping field in Venn diagram, Fig. 6.

| Protein | Function | Fold change >1.5 or < 0.67; *P* < 0.05 |
| --- | --- | --- |
| 9 proteins | | |
| Atu0946 | Dehydrogenase | WT(D) / WT(L)=1.64; M(L) / WT(L)=3.04 |
| RpsI (Atu1246) | 30S ribosomal protein S9 | WT(D) / WT(L)=1.53; M(L) / WT(L)=2.43 |
| YajC (Atu1563) | Preprotein tranlocase protein | WT(D) / WT(L)=2.04; M(L) / WT(L)=2.99 |
| RpmG (Atu1299) | 50S ribosomal protein L33 | WT(D) / WT(L)=1.52; M(L) / WT(L)=1.95 |
| Atu4600 | ABC transporter, nucleotide binding/ATPase protein | WT(D) / WT(L)=1.75; M(L) / WT(L)=2.24 |
| CyoC (Atu0140) | Cytochrome o ubiquinol oxidase subunit III | WT(D) / WT(L)=1.69; M(L) / WT(L)=2.39 |
| SoxD (Atu4068) | Sarcosine oxidase delta subunit | WT(D) / WT(L)=1.89; M(L) / WT(L)=2.34 |
| NapE (Atu4405) | Periplasmic nitrate reductase protein | WT(D) / WT(L)=1.69; M(L) / WT(L)=2.39 |
| Atu8036 | Protein YBGT-related protein | WT(D) / WT(L)=2.13; M(L) / WT(L)=2.6 |
| 3 proteins |  |  |
| Atu8154 | Plasmid stabilization system protein | WT(D) / WT(L)=0.66; M(L) / WT(L)=0.52 |
| Atu4555 | putative xylose isomerase | WT(D) / WT(L)=0.55; M(L) / WT(L)=0.54 |
| Atu3117 | putative dihydrodipicolinate synthase family protein | WT(D) / WT(L)=0.51; M(L) / WT(L)=0.56 |

**Supplementary Table S4. iTRAQ/TMT.** Chemotaxis proteins and flagellar motility proteins of *Agrobacterium fabrum*. CheA (Atu0517), Atu0515, CheD (Atu0521), CheR (Atu0518), Atu4805, CheD (Atu2618), McpA (Atu3094), McpA (Atu6132), McpG (Atu0738), McpA (Atu2360), Atu0514, McpA (Atu2173), Atu0373, MclA (Atu1912), MclA (Atu0526), McpA (Atu0387), McpA (Atu2223), Atu5442, Atu4736 and Atu3725 were not detected. WT (D): wild type (darkness); WT (L): wild type (white light); M (D): mutant (darkness); M (L): mutant (white light); Mean values of 3 biological replicates ± SE.

| Protein | Abundances | | | | Fold change >1.5 or < 0.67; *P* < 0.1 (*) or *P* < 0.05 (**) |
| --- | --- | --- | --- | --- | --- |
|  | WT (D) | WT (L) | M (D) | M (L) |  |
| CheB (Atu0519) | 117 + 40 | 115 + 15 | 106 + 28 | 85 + 8 |  |
| CheW (Atu2075) | 114 + 5 |  | 100 + 25 |  |  |
| CheW (Atu2617) | 99 + 10 | 118 + 8 | 84 + 2 | 82 + 10 |  |
| CheY (Atu0516) | 95 + 8 | 102 + 6 | 112 + 25 | 98 + 10 |  |
| CheY (Atu0520) | 114 + 2 | 113 + 11 | 80 + 3 | 87 + 8 |  |
| McpA (Atu0646) | | 133 + 36 |  | 67 + 10 |  |
| McpC (Atu0872) : methyl-accepting chemotaxis protein | 118 + 24 |  | 47 + 10 |  | M(D) / WT(D)=0.40 * |
| McpV (Atu1027) | 83 + 9 | 65 + 6 | 112 + 22 | 135 + 57 |  |

**Supplementary Table S5. iTRAQ/TMT,** flagellar assembly proteins and flagellar proteins of bacterial motility of *Agrobacterium fabrum*. FlaF (Atu0577), FlbT (Atu0578), FlgA (Atu0551), FlgB (Atu0555), FlgC (Atu0554), FlgD (Atu0579), FlgF (Atu0558), FlgG (Atu0552), FlgH (Atu0548), FlgI (Atu0550), FlhA (Atu0581), FlhB (Atu0564), FliE (Atu0553), FliF (Atu0523), FliG (Atu0563), FliI (Atu0557), FliL (Atu0547), FliM (Atu0561), FliQ (Atu0580), FliR (Atu0582), MotA (Atu0560), MotB (Atu0569) and MotD (Atu0571) were not detected. WT (D): wild type (darkness); WT (L): wild type (white light); M (D): mutant (darkness); M (L): mutant (white light); Mean values of 3 biological replicates ± SE.

| Protein | Abundances | | | | Fold change >1.5 or < 0.67; *P* < 0.1 (*) or *P* < 0.05 (**) | | | |  |
| --- | --- | --- | --- | --- | --- | --- | --- | --- | --- |
|  | WT (D) | WT (L) | M (D) | M (L) |  |  |  |  |  |
| Fla (Atu0542) | 103 + 3 | 85 + 6 | 99 + 9 | 115 + 17 |  | | | |  |
| FlaA (Atu0545) : flagella associated protein | 82 + 6 | 71 + 2 | 131 + 16 | 129 + 25 | M(D) / WT(D)=1.60 **; M(L) / WT(L)=1.82 ** | | | |  |
| FlaB (Atu0543): flagellin domain protein | 81 + 8 | 72 + 4 | 133 + 8 | 129 + 21 | M(D) / WT(D)=1.64 **; M(L) / WT(L)=1.80 * | | | |  |
| FlaD (Atu0567) | 105 + 4 | 122 + 21 | 93 + 12 | 79 + 4 |  | | | |  |
| FlgE (Atu0574) | 106 + 8 | 107 + 12 | 98 + 8 | 93 + 4 |  | | | |  |
| FlgK (Atu0575) | 104 + 8 | 114 + 16 | 105 + 27 | 86 + 2 |  | | | |  |
| FlgL (Atu0576) | | 91 + 10 |  | 109 + 11 |  | | | |  |
| FliN (Atu0562) | 99 + 8 | 86 + 2 | 134 + 36 | 114 + 5 |  | | | |  |
| FliP (Atu0546) | | 93 + 17 |  | 107 + 19 |  | | | |  |
| MotB (Atu3746) | 88 + 20 | 81 + 3 | 114 + 38 | 120 + 16 |  |  |  |  | |
| MotC (Atu0570): chemotaxis motility protein | 148 + 42 | 157 + 53 | 29 + 2 | 43 + 2 | M(D) / WT(D)=0.20 **;  M(L) / WT(L)=0.27 * | | | |  |

**Supplementary Table S6. iTRAQ/TMT,** pilus proteins and bacterial motility proteins of *Agrobacterium fabrum*. PilA (Atu3514), CtpB (Atu0223), CtpF (Atu0219), CtpG (Atu0218) and Atu4731 were not detected. WT (D): wild type (darkness); WT (L): wild type (white light); M (D): mutant (darkness); M (L): mutant (white light); Mean values of 3 biological replicates ± SE.

| Protein | Abundances | | | | Fold change >1.5 or < 0.67; *P* < 0.1 (*) or *P* < 0.05 (**) |
| --- | --- | --- | --- | --- | --- |
|  | WT (D) | WT (L) | M (D) | M (L) |  |
| CtpA (Atu0224): components of type IV pilus, pilin subunit | 94 + 5 | 79 + 7 | 111 + 33 | 121 + 9 | M(L) / WT(L)=1.53 ** |
| CtpC (Atu0222) | 82 + 20 | 118 + 13 | 78 + 6 | 82 + 5 |  |
| CtpD (Atu0221) | 99 + 6 | 97 + 6 | 135 + 24 | 103 + 10 |  |
| CtpE (Atu0220): components of type IV pilus | 175 + 23 | 130 + 13 | 58 + 15 | 70 + 3 | M (D) / WT (D)=0.33 **; M (L) / WT (L)=0.54 ** |
| Atu4732 | 93 + 12 | 99 + 4 | 101 + 3 | 101 + 14 |  |

**Supplementary Table S7. iTRAQ/TMT,** conjugation proteins of *Agrobacterium fabrum*. TraA (Atu4855), TraA (Atu6127), TraB (Atu6129), TraC (Atu5110), TraF (Atu6128), TraG (Atu5108), TraG (Atu6124), TraH (Atu6130), TrbB (Atu6041), TrbC (Atu6040), TrbD (Atu6039), TrbE (Atu6038), TrbF (Atu6034), TrbG (Atu6033), TrbH (Atu6032), TrbI (Atu6031), TrbJ (Atu6037), TrbL (Atu6035) were not detected. WT (D): wild type (darkness); WT (L): wild type (white light); M (D): mutant (darkness); M (L): mutant (white light); Mean values of 3 biological replicates ± SE.

| Protein | Abundances | | | | Fold change >1.5 or < 0.67; *P* < 0.1 (*) or *P* < 0.05 (**) |
| --- | --- | --- | --- | --- | --- |
|  | WT (D) | WT (L) | M (D) | M (L) |  |
| MobC (Atu4857): mobilization protein C | 87 + 9 | 96 + 6 | 138 + 15 | 104 + 11 | M (D) / WT (D)=1.59 ** |
| TraA (Atu5111): conjugal transfer relaxase | 147 + 22 |  | 49 + 15 |  | M (D) / WT (D)=0.33 ** |
| TraC (Atu6126): conjugal transfer protein | 69 + 6 | 67 + 6 | 161 + 13 | 133 + 13 | M (D) / WT (D)=2.34 **;  M (L) / WT (L)=2 ** |
| TraD (Atu5109): conjugal transfer protein | 85 + 13 | 87 + 1 | 133 + 14 | 113 + 5 | M (D) / WT (D)=1.56 * |

**Supplementary Table S8. iTRAQ/TMT,** virulence proteins without type IV secretion proteins of *Agrobacterium fabrum*. VirA (Atu6166), VirC1 (Atu6180), VirC2 (Atu6179), VirD1 (Atu6181), VirD2 (Atu6182), VirD3 (Atu6183), AvhD4 (Atu4858), VirD4 (Atu6184), VirD5 (Atu6185), VirE0 (Atu6188), VirE1 (Atu6189), VirE2 (Atu6190), VirE3 (Atu6191), VirE3 (Atu6186), VirF (Atu6154), VirG (Atu6178), VirK (Atu6156), MviN (Atu0347), TrlR (Atu6192), Atu6193, Atu6194, Atu6195, Atu6196 and Atu6197 were not detected. WT (D): wild type (darkness); WT (L): wild type (white light); M (D): mutant (darkness); M (L): mutant (white light); Mean values of 3 biological replicates ± SE.

| Protein | Abundances | | | | Fold change >1.5 or < 0.67; *P* < 0.1 (*) or *P* < 0.05 (**) |
| --- | --- | --- | --- | --- | --- |
|  | WT (D) | WT (L) | M (D) | M (L) |  |
| VirH1 (Atu6187) | 105 + 10 | 115 + 2 | 97 + 3 | 85 + 5 |  |
| AcvB (Atu2522) | 104 + 3 | 111 + 3 | 98 + 9 | 89 + 5 |  |

**Supplementary Table S9. iTRAQ/TMT,** type IV secretion proteins of *Agrobacterium fabrum*. VirB1 (Atu6167), AvhB2 (Atu5163), VirB2 (Atu6168), AvhB3 (Atu5164), VirB3 (Atu6169), VirB4 (Atu6170), VirB5 (Atu6171), AvhB6 (Atu5167), VirB6 (Atu6172), VirB8 (Atu6174), VirB9 (Atu6175), VirB10 (Atu6176), AvhB11 (Atu5172), VirB11 (Atu6177), Atu4858 (traG), Atu5108 (traG), Atu6124 (traG) and virD4 (Atu6184) of type IV secretion system were not detected. WT (D): wild type (darkness); WT (L): wild type (white light); M (D): mutant (darkness); M (L): mutant (white light); Mean values of 3 biological replicates ± SE.

| Protein | Abundances | | | | Fold change >1.5 or < 0.67; *P* < 0.1 (*) or *P* < 0.05 (**) |
| --- | --- | --- | --- | --- | --- |
|  | WT (D) | WT (L) | M (D) | M (L) |  |
| AvhB1 (Atu5162): type IV secretion protein | 59 + 4 | 88 + 9 | 145 + 19 | 112 + 3 | WT (L) / WT (D)=1.50 **; M (D) / WT (D)=2.46 ** |
| AvhB4 (Atu5165): type IV secretion protein | 78 + 3 | 69 + 5 | 133 + 29 | 131 + 23 | M (L) / WT (L)=1.89 * |
| AvhB5 (Atu5166) | 69 + 1 | 100 + 2 | 132 + 57 | 100 + 5 |  |
| AvhB7 (Atu5168): type IV secretion protein | 82 + 11 | 79 + 11 | 121 + 23 | 121 + 4 | M (L) / WT (L)=1.52 ** |
| VirB7 (Atu6173) | 100 + 5 |  | 133 + 21 |  |  |
| AvhB8 (Atu5169) | 89 + 19 | 96 + 13 | 100 + 20 | 104 + 9 |  |
| AvhB9 (Atu5170): type IV secretion protein | 74 + 4 | 81 + 9 | 137 + 2 | 119 + 7 | M (D) / WT (D)=1.85 ** |
| AvhB10 (Atu5171): type IV secretion protein | 74 + 5 | 66 + 5 | 136 + 1 | 134 + 4 | M (D) / WT (D)=1.84 **; M (L) / WT (L)=2.01 ** |

**Supplementary Table S10. iTRAQ/TMT,** Type VI secretion system and three toxin-immunity pairs - *Agrobacterium fabrum*. WT (D): wild type (darkness); WT (L): wild type (white light); M (D): mutant (darkness); M (L): mutant (white light); Mean values of 3 biological replicates ± SE.

| Protein | Abundances | | | | Fold change >1.5 or < 0.67; *P* < 0.1 (*) or *P* < 0.05 (**) |
| --- | --- | --- | --- | --- | --- |
|  | WT (D) | WT (L) | M (D) | M (L) |  |
| Type VI secretion system | | | | | |
| VgrG (Atu4348) | 87 + 2 | 81 + 7 | 146 + 82 | 119 + 20 |  |
| Hcp (Atu4345): putative hemolysin-coregulated protein | 114 + 9 | 126 + 9 | 76 + 1 | 74 + 4 | M (D) / WT (D)=0.67 **; M (L) / WT (L)=0.59 ** |
| ImpL (Atu4332) | 86 + 4 | 84 + 4 | 115 + 12 | 116 + 12 |  |
| ImpK (Atu4333) | 96 + 5 | 86 + 6 | 96 + 7 | 114 + 11 |  |
| ClpB (Atu4334) | 107 + 7 | 89 + 4 | 85 + 8 | 111 + 11 |  |
| Effectors of three toxin-immunity pairs of type VI secretion system | | | | | |
| Atu4350 (1-toxin) | 105 + 5 | 101 + 1 | 98 + 9 | 99 + 8 |  |
| Atu4351 (1-immunity) | 101 + 4 | 117 + 3 | 103 + 5 | 83 + 9 |  |
| Atu3640 (2-toxin) | 93 + 7 | 103 + 14 | 106 + 24 | 97 + 3 |  |
| Atu3639 (2-immunity) | 94 + 11 | 97 + 2 | 113 + 15 | 103 + 8 |  |
| Atu4347 (3-toxin): putative peptidoglycan amidase | 114 + 2 | 123 + 7 | 85 + 1 | 77 + 3 | M (L) / WT (L)=0.63 ** |
| Atu4346 (3-immunity) | 90 + 8 | 99 + 5 | 121 + 7 | 101 + 8 |  |
